# Postoperative change in the joint-line convergence angle is associated with inaccurate correction in open-wedge high tibial osteotomy

**DOI:** 10.1186/s13018-023-04248-9

**Published:** 2023-10-13

**Authors:** Young Mo Kim, Yong Bum Joo, Young Cheol Park, Seung-ho Lee, Ju-Ho Song

**Affiliations:** 1 Department of Orthopedic Surgery, Chungnam National University Hospital, Chungnam National University College of Medicine, Daejeon, Republic of Korea; 2 Department of Orthopedic Surgery, Chungnam National University Sejong Hospital, Chungnam National University College of Medicine, Sejong, 30099 Republic of Korea

**Keywords:** Open-wedge high tibial osteotomy, Joint-line convergence angle, Alignment correction, Soft tissue laxity

## Abstract

**Objective:**

Accurate correction is a prerequisite for the favorable outcomes of open-wedge high tibial osteotomy (OWHTO). However, previous studies have reported disappointing results regarding correction accuracy despite the use of intra-operative navigation, which implies that a certain factor other than bony components is involved in the inaccurate correction (mainly overcorrection). The joint-line convergence angle (JLCA) can represent soft tissue effects in OWHTO. This study tried to determine whether the postoperative change in the JLCA (∆JLCA) led to inaccurate correction.

**Methods:**

Medical records of 78 OWHTO patients from 2005 to 2021 were retrospectively reviewed. The hip–knee–ankle angle (HKA) was measured with a positive value indicating varus alignment. Inaccurate correction was defined as postoperative HKA < − 3°. The JLCA was measured before and 6 months after surgery on long-standing hip-to-ankle radiographs, and ∆JLCA was defined as the difference between the preoperative and 6-month postoperative JLCAs. ∆JLCA was compared between the accurate correction group and the inaccurate correction group, and a receiver operating characteristic (ROC) curve was used to obtain the cutoff ∆JLCA at which the sensitivity and the specificity for inaccurate correction were maximized. Clinical outcomes were also compared between the groups using the knee injury and osteoarthritis outcome score (KOOS) at final follow-up (60.9 ± 53.3 months postoperatively).

**Results:**

Of the 78 patients, inaccurate correction was noted in 10 patients. The overall preoperative and postoperative HKAs were 7.0 ± 3.1° and − 0.4 ± 1.5°, respectively. The accurate correction group and the inaccurate correction group had a difference in ∆JLCA (*p* = 0.010). However, no significant difference was found in the preoperative HKA (*p* = 0.529). An ROC curve showed that the cutoff ∆JLCA was 1.9°. In the patients having ∆JLCA ≥ 1.9°, the mean JLCA was 4.9 ± 1.6° preoperatively and 1.7 ± 1.2° postoperatively. In the other patients having ∆JLCA < 1.9°, the mean preoperative and postoperative JLCA were 2.5 ± 1.8° and 2.3 ± 1.8°, respectively. The difference in the preoperative JLCA was significant (*p* < 0.001). The postoperative KOOS subscales did not differ according to correction accuracy.

**Conclusion:**

Inaccurate correction in OWHTO, specifically valgus overcorrection, is associated with large ∆JLCA which represents the postoperative change of soft tissue effects. Overcorrection should be checked in cases of large preoperative JLCAs.

## Introduction

Open-wedge high tibial osteotomy (OWHTO) realigns the limb alignment and alleviates the load on the medial compartment [1–4]. Its well-documented efficacy depends on the accurate correction of the limb alignment [5–7]. Preoperative planning is focused on the osteotomy gap or the medial proximal tibial angle (MPTA), aiming for a hip–knee–ankle angle (HKA) of 183°–186° [8–10]. Despite advanced surgical techniques and intra-operative navigation in OWHTO, previous studies have reported that correction accuracy was unexpectedly low [6, 11]. Several studies attributed discrepancies between preoperative planning and postoperative alignment to soft tissue effects that were not reflected in correction parameters, such as the osteotomy gap and the MPTA [7, 12, 13].

The joint-line convergence angle (JLCA) represents soft tissue tension of the medial side of the knee joint with varus deformity [14, 15]. Because intra-articular pressure is affected by the soft tissue tension, OWHTO is performed after releasing the superficial medial collateral ligament (sMCL) [16]. As a result, the JLCA changes postoperatively, which alters the whole limb alignment. When the JLCA became parallel, the limb alignment moves to more valgus than preoperatively planned [12, 17].

Seo et al. measured the tension of the medial structure in OWHTO, concluding that the medial laxity after releasing the sMCL was restored with the osteotomy site being opened [18]. If so, the postoperative change in the JLCA (∆JLCA) would reach an end or be reversed when an opening gap is large and the release of the sMCL is insufficient [19]. ∆JLCA is worth investigating because it could be associated with both overcorrection and undercorrection. Therefore, the present study tried to determine whether ∆JLCA led to inaccurate correction. It was hypothesized that ∆JLCA would be associated with inaccurate correction.

## Methods

Medical records of patients who underwent OWHTO from 2005 to 2021 were retrospectively reviewed after approval was obtained from our institutional review board. OWHTO was indicated in physically active patients who aged < 65 years and complained of medial-sided walking pain that did not respond to conservative treatments. The contraindications included medial compartment arthritis of Ahlbӓck grade ≥ 3, flexion contracture more than 10°, active inflammatory arthritis, and symptomatic osteoarthritis of the lateral compartment and the patellofemoral joint. The severity of varus deformity was evaluated before surgery using long-standing hip-to-ankle radiographs, and correction accuracy was checked 6 months after surgery. Clinical outcomes were assessed using the knee injury and osteoarthritis outcome score (KOOS) at final follow-up (60.9 ± 53.3 months postoperatively).

The inclusion criteria of the present study were (1) primary OWHTO, (2) follow-up duration ≥ 6 months, and (3) 6-month postoperative long-standing hip-to-ankle radiograph to measure the JLCA. Patients were excluded if they had complications that could affect correction accuracy, such as metal failures and collapse of the osteotomy site. Of the 80 patients undergoing the index surgery during the study period, 2 patients who did not take postoperative radiographs were excluded. No patients were excluded owing to metal failures or collapse of the osteotomy site. Accordingly, 78 patients met the inclusion criteria.

### Surgical technique

All OWHTOs were performed by a single senior surgeon. The correction angle was assessed on a long-standing hip-to-ankle radiograph using the Miniaci method [20]. The target weight-bearing point was Fujisawa point that corresponded to the HKA of 3° valgus [21, 22]. The correction amount was adjusted based on the severity of degenerative changes in each compartment. A 4–5 cm vertical incision was made on the anteromedial side of the tibia. The distal fibers of the sMCL and the pes anserinus tendon were released. Two guide wires were inserted with the visual assistance of an image intensifier, and osteotomy was performed, leaving 5–10 mm of the lateral cortex intact as a hinge. The ascending part of biplanar osteotomy was made behind the patella tendon, at an angle of around 110° to the previous osteotomy line. Then, the osteotomy site was opened using a laminar spreader and fixed with a locking anatomic plate. Weight-bearing was gradually allowed from toe-touch to full weight-bearing at 6–8 weeks postoperatively. The locking plate was removed at around 12 months postoperatively after verifying solid bone union at the osteotomy gap.

### Study design

The JLCA was defined as the angle between the two articular lines of the distal femur and proximal tibia, with positive values indicating varus convergence. The JLCA was measured before and 6 months after surgery on long-standing hip-to-ankle radiographs. ∆JLCA was defined as the difference between the preoperative and 6-month postoperative JLCAs.

The hip–knee–ankle angle (HKA) was measured with a positive value indicating varus alignment. Inaccurate correction was defined as postoperative HKA < − 3°. ∆JLCA was compared between the accurate correction group and the inaccurate correction group, and a receiver operating characteristic (ROC) curve was used to obtain the cutoff ∆JLCA at which the sensitivity and the specificity for inaccurate correction were maximized.

### Statistical analysis

The following factors were assessed to identify risk factors for inaccurate correction: age, sex, body mass index, preoperative HKA, preoperative and postoperative JLCAs, and ∆JLCA. Comparative analyses were performed between the accurate correction group and the inaccurate correction group. Continuous variables were compared using *t* test for normal distribution and Mann–Whitney *U* test for non-normal distribution. Data normality was tested using Shapiro–Wilk test. Categorical variables were analyzed using Chi-square test if the expected value of the cell was ≥ 5 in at least 80% of the cells; otherwise, Fisher exact test was applied. All statistical analyses were conducted using R software version 4.1.1 (R foundation for Statistical Computing, Vienna, Austria), with a *p* value < 0.05 considered statistically significant.

## Results

Seventy-eight patients with a mean age of 52.0 ± 4.8 years (range, 35–63 years) were followed for a mean duration of 60.9 ± 53.3 months (range, 9–102 months). Of these, inaccurate correction was noted in 10 patients. The overall preoperative and postoperative HKAs were 7.0 ± 3.1° and − 0.4 ± 1.5°, respectively. The accurate correction group and the inaccurate correction group had a difference in ∆JLCA (*p* = 0.010). However, no significant difference was found in the preoperative HKA (*p* = 0.529). The patient characteristics between the groups are summarized in Table 1.

**Table 1:** Patient characteristics

	Overall	Accurate correction (*N* = 68)	Inaccurate correction (*N* = 10)	*p* value
Age, year^﻿a﻿^	52.0 ± 4.8	52.0 ± 4.8	52.0 ± 4.3	0.897
Male/Female, *n*	18/60	17/51	1/9	0.438
BMI, kg/m^2a^	26.9 ± 3.8	27.1 ± 3.7	25.4 ± 4.0	0.185
Follow-up duration, month^﻿a﻿^	60.9 ± 53.3	61.2 ± 52.8	59.4 ± 60.0	0.973
HKA, deg^b^				
Preoperative^﻿a﻿^	7.0 ± 3.1	6.9 ± 3.1	7.6 ± 2.7	0.529
Postoperative^﻿a﻿^	− 0.4 ± 1.5	− 0.3 ± 1.6	− 4.5 ± 0.8	< 0.001
JLCA, deg^c^				
Preoperative^﻿a﻿^	3.2 ± 2.0	3.1 ± 2.0	4.1 ± 1.9	0.105
Postoperative^﻿a﻿^	2.1 ± 1.7	2.2 ± 1.7	1.7 ± 1.1	0.423
∆JLCA^﻿a﻿^	1.0 ± 1.8	0.8 ± 1.7	2.4 ± 1.8	0.010
KOOS				
Pain^﻿a﻿^	81.4 ± 12.7	80.8 ± 13.0	85.4 ± 11.4	0.662
Symptoms^﻿a﻿^﻿	80.7 ± 15.9	79.7 ± 16.5	88.4 ± 8.9	0.340
ADL^﻿a﻿^	87.7 ± 13.4	86.9 ± 13.9	93.8 ± 6.7	0.511
Sports/Rec^﻿a﻿^	53.8 ± 31.3	52.4 ± 32.6	63.8 ± 19.3	0.478
QoL^﻿a﻿^	68.8 ± 19.7	68.2 ± 19.6	73.4 ± 23.0	0.609

*BMI* body mass index, *HKA* hip–knee–ankle angle, *JLCA* joint-line convergence angle, *KOOS* Knee Injury and Osteoarthritis Outcome, *ADL* activities in daily living, *Sports/Rec* sports and recreational function, *QoL* quality of life

^a^Data are reported as mean ± SD unless otherwise indicated

^b^Positive values indicate varus alignment, whereas negative values indicate valgus alignment

^c^Positive values indicate varus convergence, whereas negative values indicate valgus convergence

### Evaluation of ∆JLCA and inaccurate correction

**Fig. 1:**
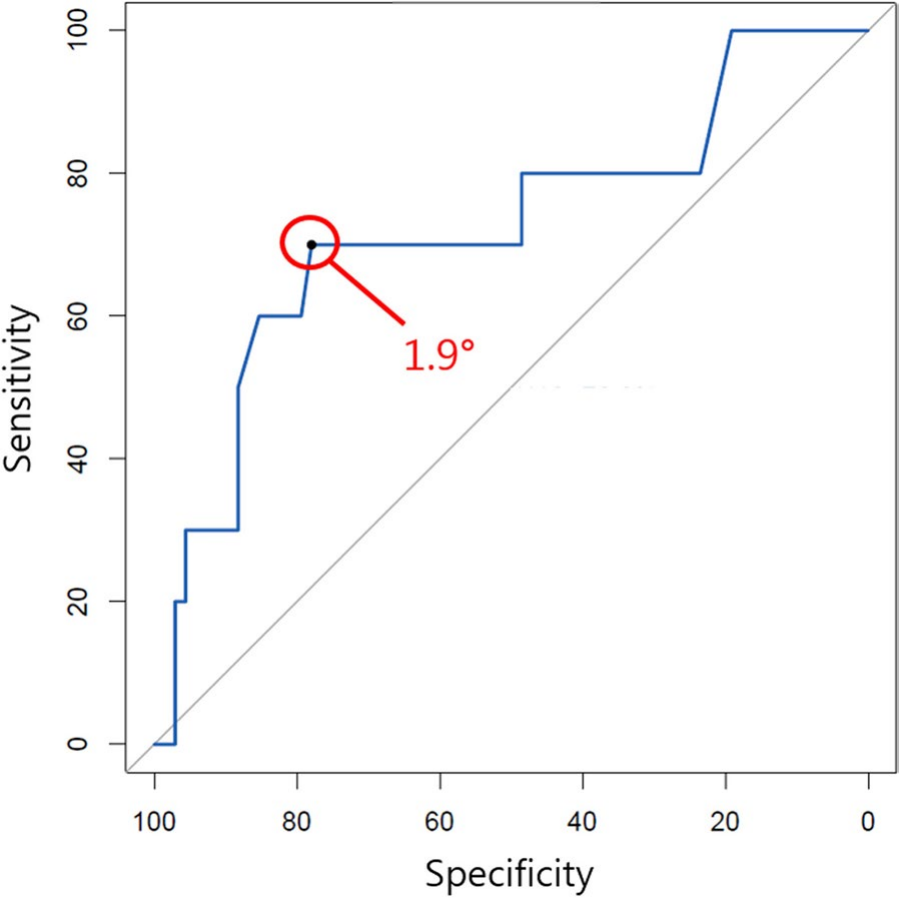
Receiver operating characteristic curve for inaccurate correction showed that the cutoff ∆JLCA was 1.9° with the area under curve being 0.72 (sensitivity, 70.0%; specificity, 77.9%)

An ROC curve for inaccurate correction showed that the cutoff ∆JLCA was 1.9° with the area under curve being 0.72 (sensitivity, 70.0%; specificity, 77.9%; Fig. 1). In the patients having ∆JLCA ≥ 1.9°, the mean JLCA was 4.9 ± 1.6° preoperatively and 1.7 ± 1.2° postoperatively. In the other patients having ∆JLCA < 1.9°, the mean preoperative and postoperative JLCA were 2.5 ± 1.8° and 2.3 ± 1.8°, respectively (Table 2). The difference in the preoperative JLCA was significant (*p* < 0.001). The postoperative KOOS subscales did not differ according to correction accuracy (Table 1).

**Table 2:** Analyses according to ∆JLCA

JLCA	∆JLCA ≥ 1.9° (*N* = 22)	∆JLCA < 1.9° (*N* = 56)	*p* value
Preoperative JLCA^﻿a﻿^	4.9 ± 1.6	2.5 ± 1.8	< 0.001
Postoperative JLCA^﻿a﻿^	1.7 ± 1.2	2.3 ± 1.8	0.125

JLCA, joint-line convergence angle

^a^Data are reported as mean ± SD unless otherwise indicated

## Discussion

The most important findings of the present study were that 1) the postoperative decrease of the JLCA (∆JLCA of 1.9°) could lead to valgus overcorrection, and 2) the preoperative JLCA differed significantly according to ∆JLCA. Therefore, overcorrection should be checked when a large JLCA was noted preoperatively.

The amount of ∆JLCA is hard to predict before performing OWHTO. Albeit delicate preoperative planning and the application of intra-operative navigation, the unexpectedly large ∆JLCA often leads to overcorrection. Lee et al. proved that change in soft tissue laxity from before to after OWHTO correlated with both correction amount and correction error [12]. The question is how much ∆JLCA would be associated with inaccurate correction. The present study showed that ∆JLCA of 1.9° was associated with overcorrection and that amount of ∆JLCA occurred when the mean preoperative JLCA was around 5°.

According to a study measuring the joint space width at different time points after OWHTO, the medial joint space width increased 3 months after surgery and the lateral joint space width decreased immediately [15]. Given that lateral joint space narrowing was not associated with the cartilage grades of the medial compartment, the immediate decrease of the lateral joint width implies ∆JLCA which is a representative parameter of soft tissue effects. This change is also the case of double varus syndrome coined by Noyes [23], in which varus deformity is aggravated by the slackness of the lateral soft tissue. Thus, the postoperative decrease of JLCA indicates the change of the lateral soft tissue laxity after the osteotomy gap is opened and the varus aggravation is alleviated.

No consensus has been made on the appropriate extent of medial release in OWHTO. Some studies reported that the release or transection of the sMCL did not increase valgus laxity [18, 24]. On the other hand, another study argued that the sMCL release should be kept to a minimum in cases of small wedge sizes [25]. In the present study, no patient showed the valgus convergence of the joint line (negative JLCA). However, large preoperative JLCAs were more likely to decrease larger than 1.9° and lead to overcorrection. Weiping et al. came to similar conclusion that a higher percentage of patients with preoperative JLCA ≤ 6° achieved optimal postoperative JLCA [26]. If the correction amount is moderate and medial tightness is relieved enough before osteotomy, it should be the slackness of the lateral soft tissue that primarily affects ΔJLCA. However, to predict ∆JLCA in detail, the consistent method and extent of medial release should be established, and individual medial tightness has to be considered. Future studies are expected to give surgeons a predictive formula for ∆JLCA.

Some limitations should be noted. First, all OWHTOs were performed by a single senior surgeon. There are several ways to address the medial soft tissue before osteotomy. However, this study could not evaluate the effect of each method on correction accuracy. Second, there might be unrevealed factors influencing ∆JLCA. Of those who had large preoperative JLCAs, some patients showed large ∆JLCA, whereas the others did not. We assumed that different medial tightness of each patient would contribute to the difference. However, it was impossible to prove the assumption in this study. Third, the retrospective design did not allow us to assess the difference between a target alignment and an actual postoperative alignment in each case. Instead, inaccurate correction was defined based on the widely accepted target range of the alignment [9, 27]. Fourth, the sample size could not be tested accurately because of the retrospective design of the study, and there might have been a significant difference in the KOOS subscales if the sample size had been large enough.

## Conclusion

Inaccurate correction in OWHTO, specifically valgus overcorrection, is associated with large ∆JLCA which represents the postoperative change of soft tissue effects. Overcorrection should be checked in cases of large preoperative JLCAs.

## Data Availability

The datasets used and/or analyzed during the current study are available from the corresponding author on reasonable request.

## References

[1] Song J-H, Bin S-I, Kim J-M, Lee B-S, Choe J-S, Cho H-K (2022). Insufficient correction and preoperative medial tightness increases the risk of varus recurrence in open-wedge high tibial osteotomy. Arthroscopy.

[2] Otsuki S, Ikeda K, Wakama H, Okuno N, Okamoto Y, Okayoshi T (2020). Preoperative flexion contracture is a predisposing factor for cartilage degeneration at the patellofemoral joint after open wedge high tibial osteotomy. Knee Surg Relat Res.

[3] Staubli AE, De Simoni C, Babst R, Lobenhoffer P (2003). TomoFix: a new LCP-concept for open wedge osteotomy of the medial proximal tibia–early results in 92 cases. Injury.

[4] Jeong HW, Song YS, Kim JS, Nam HS, Lee WW, Lee YS (2023). Serial quantitative assessment of load redistribution after medial open-wedge high tibial osteotomy. Orthop J Sports Med.

[5] Akamatsu Y, Mitsugi N, Mochida Y, Taki N, Kobayashi H, Takeuchi R (2012). Navigated opening wedge high tibial osteotomy improves intraoperative correction angle compared with conventional method. Knee Surg Sports Traumatol Arthrosc.

[6] Van den Bempt M, Van Genechten W, Claes T, Claes S (2016). How accurately does high tibial osteotomy correct the mechanical axis of an arthritic varus knee? A systematic review. Knee.

[7] Tsuji M, Akamatsu Y, Kobayashi H, Mitsugi N, Inaba Y, Saito T (2020). Joint line convergence angle predicts outliers of coronal alignment in navigated open-wedge high tibial osteotomy. Arch Orthop Trauma Surg.

[8] Bouguennec N, Mergenthaler G, Gicquel T, Briand C, Nadau E, Pailhé R (2020). Medium-term survival and clinical and radiological results in high tibial osteotomy: Factors for failure and comparison with unicompartmental arthroplasty. Orthop Traumatol Surg Res.

[9] Hernigou P, Medevielle D, Debeyre J, Goutallier D (1987). Proximal tibial osteotomy for osteoarthritis with varus deformity. A ten to thirteen-year follow-up study. J Bone Joint Surg Am.

[10] Jiang X, Li B, Xie K, Ai S, Hu X, Gao L (2023). Lateral tibial intercondylar eminence is a reliable reference for alignment correction in high tibial osteotomy. Knee Surg Sports Traumatol Arthrosc.

[11] So S-Y, Lee S-S, Jung EY, Kim JH, Wang JH (2020). Difference in joint line convergence angle between the supine and standing positions is the most important predictive factor of coronal correction error after medial opening wedge high tibial osteotomy. Knee Surg Sports Traumatol Arthrosc.

[12] Lee D-H, Park S-C, Park H-J, Han S-B (2016). Effect of soft tissue laxity of the knee joint on limb alignment correction in open-wedge high tibial osteotomy. Knee Surg Sports Traumatol Arthrosc.

[13] Lee DK, Wang JH, Won Y, Min YK, Jaiswal S, Lee BH (2020). Preoperative latent medial laxity and correction angle are crucial factors for overcorrection in medial open-wedge high tibial osteotomy. Knee Surg Sports Traumatol Arthrosc.

[14] Park J-G, Kim J-M, Lee B-S, Lee S-M, Kwon O-J, Bin S-I (2020). Increased preoperative medial and lateral laxity is a predictor of overcorrection in open wedge high tibial osteotomy. Knee Surg Sports Traumatol Arthrosc.

[15] Lee S-M, Bin S-I, Kim J-M, Lee B-S, Suh KT, Song J-H (2021). Joint space width increases medially and decreases laterally at different time points after medial open-wedge high tibial osteotomy. Arthroscopy.

[16] Agneskirchner JD, Hurschler C, Wrann CD, Lobenhoffer P (2007). The effects of valgus medial opening wedge high tibial osteotomy on articular cartilage pressure of the knee: a biomechanical study. Arthroscopy.

[17] Na YG, Lee BK, Choi JU, Lee BH, Sim JA (2021). Change of joint-line convergence angle should be considered for accurate alignment correction in high tibial osteotomy. Knee Surg Relat Res.

[18] Seo S-S, Kim C-W, Seo J-H, Kim D-H, Lee C-R (2016). Does superficial medial collateral ligament release in open-wedge high tibial osteotomy for varus osteoarthritic knees increase valgus laxity?. Am J Sports Med.

[19] Shim SJ, Jeong HW, Kim S, Park Y-G, Lee YS (2022). Factors associated with unfavorable radiological outcomes after opening-wedge high tibial osteotomy for varus knees. Orthop J Sports Med.

[20] Miniaci A, Ballmer FT, Ballmer PM, Jakob RP (1989). Proximal tibial osteotomy. A new fixation device. Clin Orthop Relat Res.

[21] Takeuchi R, Ishikawa H, Aratake M, Bito H, Saito I, Kumagai K (2009). Medial opening wedge high tibial osteotomy with early full weight bearing. Arthroscopy.

[22] Kubota M, Ohno R, Sato T, Yamaguchi J, Kaneko H, Kaneko K (2019). The medial proximal tibial angle accurately corrects the limb alignment in open-wedge high tibial osteotomy. Knee Surg Sports Traumatol Arthrosc.

[23] Noyes FR, Barber-Westin SD, Hewett TE (2000). High tibial osteotomy and ligament reconstruction for varus angulated anterior cruciate ligament-deficient knees. Am J Sports Med.

[24] Kim J-H, Ryu DJ, Lee S-S, Jang SP, Park JS, Kim WJ (2022). Does transection of the superficial MCL during HTO result in progressive valgus instability? [Formula: see text]. Am J Sports Med.

[25] Pape D, Duchow J, Rupp S, Seil R, Kohn D (2006). Partial release of the superficial medial collateral ligament for open-wedge high tibial osteotomy. A human cadaver study evaluating medial joint opening by stress radiography. Knee Surg Sports Traumatol Arthrosc.

[26] Ji W, Luo C, Zhan Y, Xie X, He Q, Zhang B (2019). A residual intra-articular varus after medial opening wedge high tibial osteotomy (HTO) for varus osteoarthritis of the knee. Arch Orthop Trauma Surg.

[27] El-Azab HM, Morgenstern M, Ahrens P, Schuster T, Imhoff AB, Lorenz SGF (2011). Limb alignment after open-wedge high tibial osteotomy and its effect on the clinical outcome. Orthopedics.

